# Supplementary figures and images for: Identification of the whole genome of alternative splicing and RNA-binding proteins involved in nintedanib-induced apoptosis in gastric cancer cells
Source: PeerJ. 2024 Dec 23;12:e18697. doi: 10.7717/peerj.18697 (PMC11670762; doi:10.7717/peerj.18697)

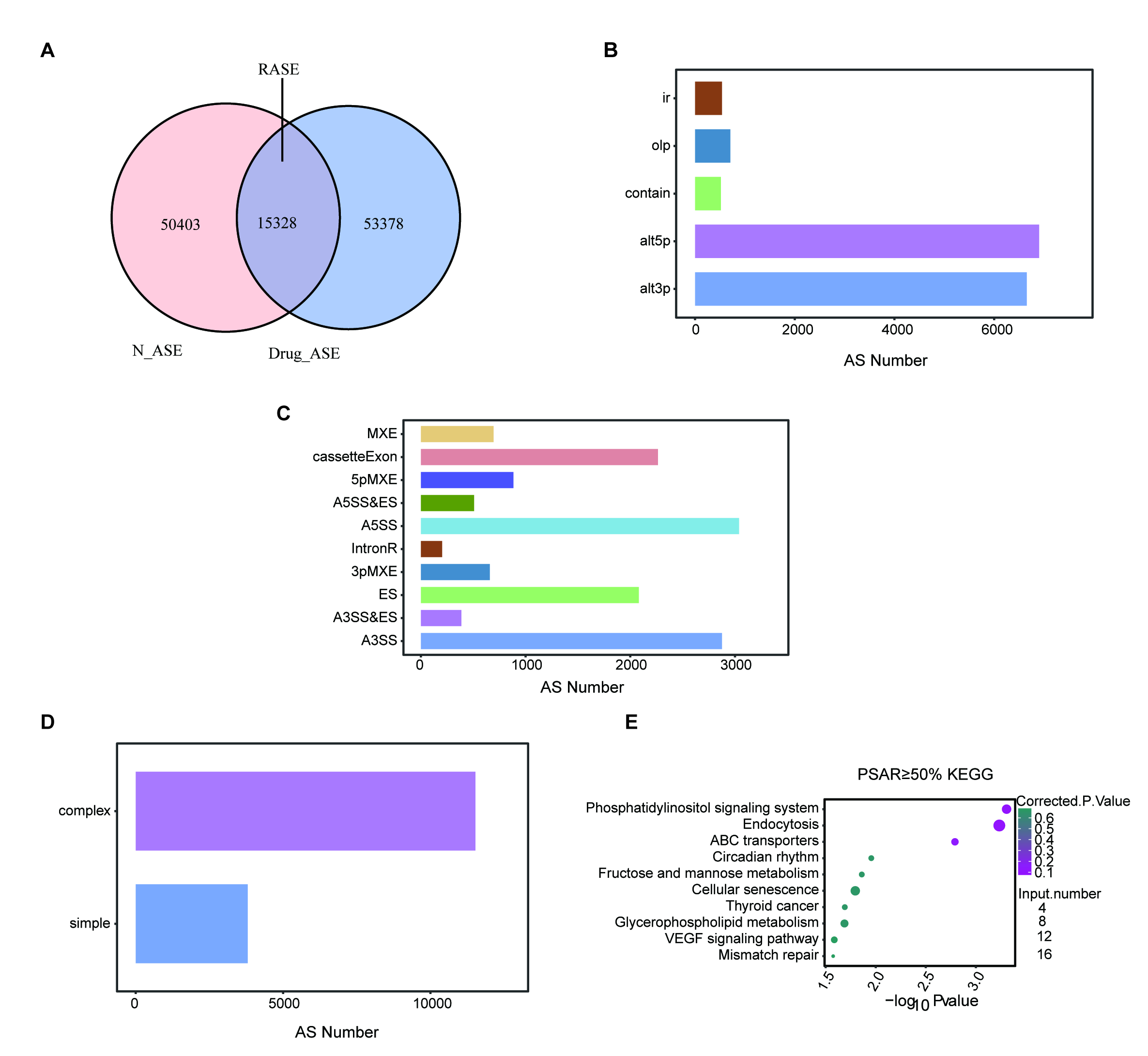

Supplement: Supplemental Information 1 — (A) A Venn diagram illustrating all splicing events and differential events detected in the normal control group and the drug treatment group. (B) Five types of AS events identified by SUVA include the predominant RAS event types, namely alt5p and alt3p events. (C) The bar chart displays the total number of splicing events identified by SUVA analysis and those obtained by conventional methods.(D) Mapping all splicing events identified by SUVA to classical splicing events, A5SS, cassette-Exon, ES, and A3SS are the most prevalent alternative splicing events.(E) KEGG enrichment analysis was conducted on genes associated with the splicing events depicted in fig1E. [file peerj-12-18697-s001.tif]

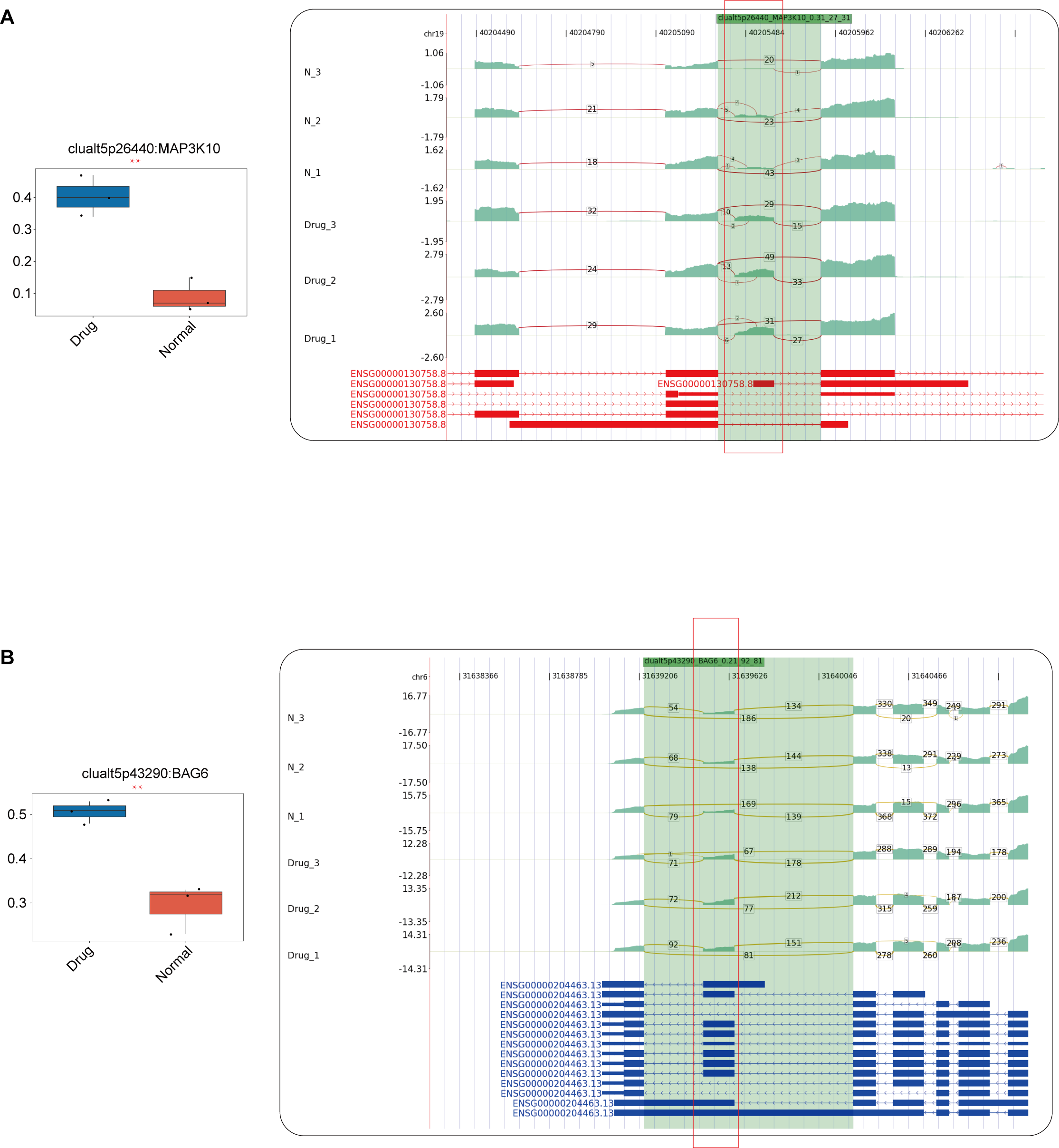

Supplement: Supplemental Information 2 — (A-B) The reads plot illustrates apoptosis-related genes: clualt5p26440:MAP3K10 and clualt5p43290:BAG6. [file peerj-12-18697-s002.tif]

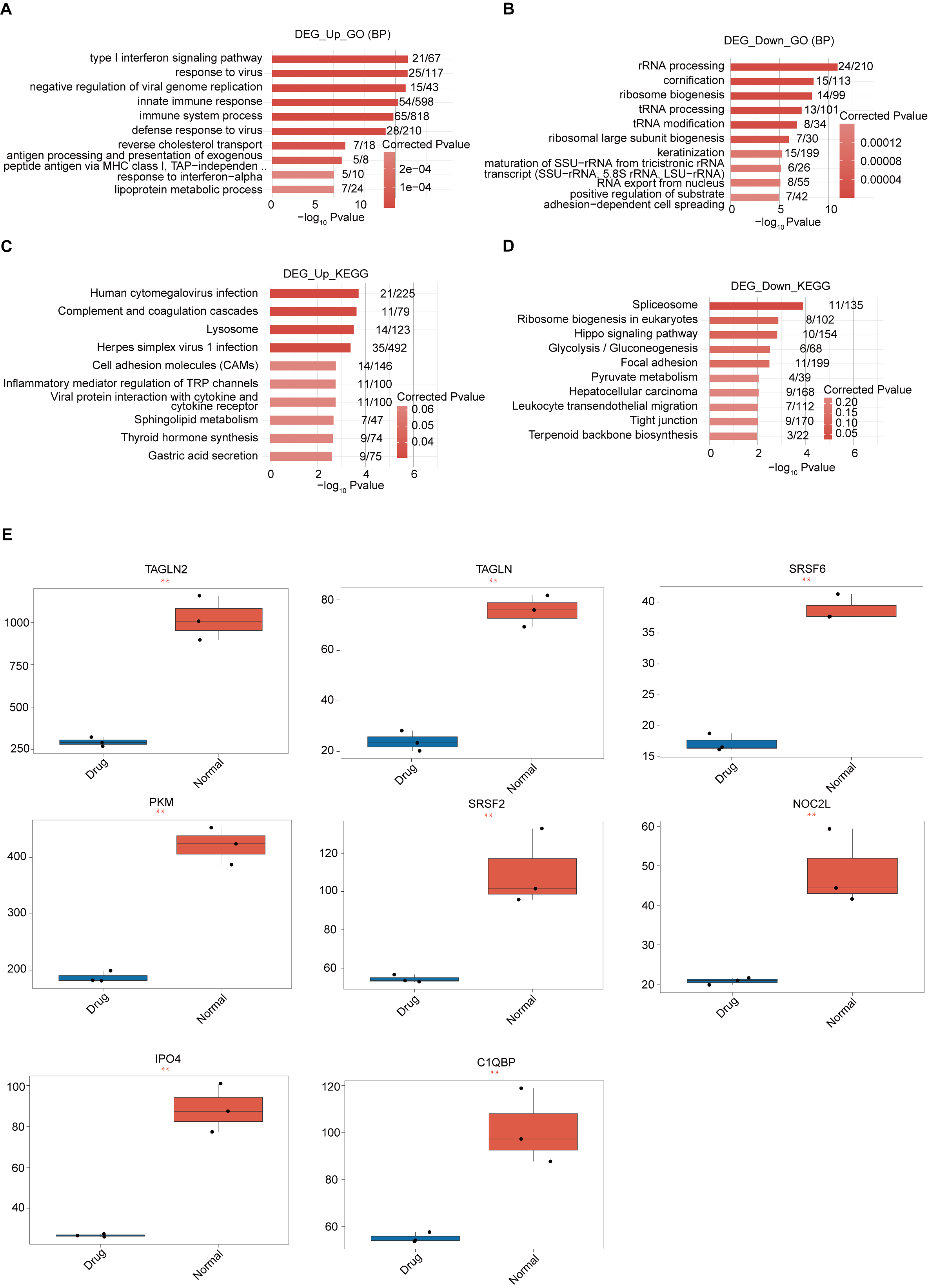

Supplement: Supplemental Information 3 — (A) GO functional enrichment analysis was conducted on all upregulated DERBPs between Drug and Normal samples, which significantly enriched biological pathways such as type I interferon signaling pathway, innate immune response, and immune system process. (B) GO functional enrichment analysis was performed on all downregulated DERBPs between Drug and Normal samples, revealing significant enrichment in biological pathways including rRNA processing, ribosome biogenesis, tRNA processing, and tRNA modification. (C) KEGG functional enrichment analysis was carried out on upregulated DERBPs, revealing enrichment in biological pathways such as Cell adhesion molecules (CAMs) and Inflammatory mediator regulation of TRP channels. (D) KEGG functional enrichment analysis was conducted on downregulated DERBPs, showing enrichment in biological pathways such as Spliceosome, Ribosome biogenesis in eukaryotes, and Hippo signaling pathway. (E) The expression levels of DERBPs in fig3 E are as follows: TAGLN2, TAGLN, SRSF6, PKM, SRSF2, NOC2L, IPO4, C1QBP. [file peerj-12-18697-s003.tif]
